# Biominerals and waxes of *Calamagrostis epigejos* and *Phragmites australis* leaves from post-industrial habitats

**DOI:** 10.1007/s00709-017-1179-8

**Published:** 2017-11-16

**Authors:** Ewa Talik, Adam Guzik, Eugeniusz Małkowski, Gabriela Woźniak, Edyta Sierka

**Affiliations:** 1 Institute of Physics, University of Silesia in Katowice, Uniwersytecka 4, 40-007 Katowice, Poland; 2 Department of Plant Physiology, Faculty of Biology and Environmental Protection, University of Silesia in Katowice, Jagiellońska 28, 40-032 Katowice, Poland; 3 Department of Botany and Nature Protection, Faculty of Biology and Environmental Protection, University of Silesia in Katowice, Jagiellońska 28, 40-032 Katowice, Poland

**Keywords:** *Calamagrostis epigejos*, *Phragmites australis*, Biomineralization, Calcium carbonate, Calcium oxalate, Phytoliths, Mineralized plant waxes

## Abstract

Vascular plants are able to conduct biomineralization processes and collect synthesized compounds in their internal tissues or to deposit them on their epidermal surfaces. This mechanism protects the plant from fluctuations of nutrient levels caused by different levels of supply and demand for them. The biominerals reflect both the metabolic characteristics of a vascular plant species and the environmental conditions of the plant habitat. The SEM/EDX method was used to examine the surface and cross-sections of the *Calamagrostis epigejos* and *Phragmites australis* leaves from post-industrial habitats (coal and zinc spoil heaps). The results from this study have showed the presence of mineral objects on the surfaces of leaves of both grass species. The calcium oxalate crystals, amorphous calcium carbonate spheres, and different silica forms were also found in the inner tissues. The high variety of mineral forms in the individual plants of both species was shown. The waxes observed on the leaves of the studied plants might be the initializing factor for the crystalline forms and structures that are present. For the first time, wide range of crystal forms is presented for *C. epigejos*. The leaf samples of *P. australis* from the post-industrial areas showed an increased amount of mineral forms with the presence of sulfur.

## Introduction

Many plant species are able to form minerals in their tissues through a complicated process called biomineralization (Skinner and Jahren 2003; Franceschi and Nakata 2005). Minerals usually are stored inside tissues; however, biomineral particles can also be observed on the surface of plant organs. The biominerals reflect both the metabolic characteristics of a vascular plant species and the environmental conditions of the plant habitat. There are three basic types of compounds, which mineralize in higher plants: calcium oxalate, calcium carbonate, and silica (Franceschi and Nakata 2005; Gal et al. 2012; He et al. 2014, 2015).

Calcium is an essential mineral element classified as macronutrient since its concentration in plant tissues ranges from 5 to 50 g/kg dry weight (DW) depending on the growing conditions, plant species, and plant organ (Mengel et al. 2001; Hawkesford et al. 2012; Bloom and Smith 2015). In the soil, it occurs in various primary minerals such as phosphates, carbonates, or calcium-bearing Al silicates (Mengel et al. 2001). Calcium movement from the soil to the root surface proceeds in soil solution mainly by mass flow and root interception (Jungk 2002). Then calcium is absorbed and subsequently moved across the root to xylem by both apoplastic and symplastic pathways. The apoplastic pathway is relatively non-selective, the result being that accumulation of calcium in shoots can frequently exceed the demand of plants particularly if concentration of calcium in soil is high (White and Broadley 2003). To avoid the toxic effect of calcium, it is precipitated in the form of calcium salts such as oxalate, phosphate, sulfate, carbonate, tartrate, citrate, and silicate (He et al. 2012 and literature therein). The predominant salt that was found in a large number of plant species is calcium oxalate (CaOx) (Bouropoulos et al. 2001; Franceschi and Nakata 2005; Nakata 2012). This calcium salt may occur in plants in a wide range of morphologies which can be classified into one of five categories: crystal sand, raphides, druses, styloids, and prismatic crystals (McConn and Nakata 2002; Franceschi and Nakata 2005). Whereas calcium oxalate crystals are well examined in dicotyledons (Franceschi and Nakata 2005; Guo Xin Xu et al. 2011), there is still a paucity of data on the presence, structure, and formation of this mineral in monocotyledons (Lersten 1983; Prychid and Rudall 1999). Particularly *Poaceae* family seems to be poorly investigated and only a few species were examined so far (Prychid and Rudall 1999; Liu et al. 2012). Also, the presence of other minerals is poorly investigated in this family. For the time being, there are few reports on biomineralization in common reed (*Phragmites australis*) (Liu et al. 2012), whereas, to the best of our knowledge, there no reports on Ca crystal formation in *Calamagrostis epigejos.*

Silicon is not essential element for plants but it is treated as beneficial element (Broadley et al. 2012), and it is generally accepted that silicon has a number of beneficial effects on plant growth (Ma and Yamaji, 2006; Adrees et al. 2015). In the soil, it is the most abundant element present in a large number of minerals (Kabata-Pendias 2011). In soil solution, the prevailing soluble form of silicon is orthosilicic acid Si(OH)_4_ (Kabata-Pendias 2011; Broadley et al. 2012) which concentration remains at a constant level at pH range from 3 to 7 (Mengel et al. 2001; Broadley et al. 2012). Plants take up silicon from soil as orthosilicic acid Si(OH)_4_. Once it is absorbed by roots, it is translocated to the shoot in the xylem. Above 90% of silicon taken up by the roots is translocated to above ground parts of plant, the result being that the concentration of this element in shoots is invariably higher than in roots (Broadley et al. 2012). In most plants, Si is deposited as amorphous silica (SiO_2_-nH_2_O), mainly in the cell walls (Pilon-Smits et al. 2009; Broadley et al. 2012). However, Ma and Yamai (2006) proposed that in roots and culms, Si exists as silicic acid Si(OH)_4_, whereas in leaf blades and leaf sheath silicic acid mainly polymerizes forming silica gel [SiO_2_·nH_2_O], thus a form of silicon in a plant depends on type of tissue. Schaller et al. (2013) confirmed this in *Phragmites australis*. Most of absorbed silicon (90%) is converted to variety of silicon-cellulose structures and phytoliths. Phytoliths are produced in cells without a lot of energy, by the polymerization of silicic acid when its concentration exceeds 2 mM (Piperno 2006). It was proved that monocotyledons accumulate higher concentration of silicon (10–15%) than dicotyledons (< 0.5%) (Pilon-Smits et al. 2009).

Besides two mentioned elements, Ca and Si, in biomineralization process other elements can take part, particularly if plants grow in disturbed habitats. Sarret et al. (2007) found that tobacco plants which grew in hydroponic culture supplemented with toxic concentrations of Cd or Zn contained calcium carbonate crystals with admixture of both heavy metals. Moreover, it was documented that different plant species growing on highly Zn-contaminated substrates of tailings formed Zn silicates. It was proposed that formation of such mineral phases could be used by plants to detoxify surplus of Zn (Neumann and de Figueiredo 2002; De Giudici et al. 2015; Medas et al. 2015). Other elements are also present in mineral structures associated with plant tissues, e.g., K, Fe, Mn, Al, Ti, Sr, and Ba, indicating the divers chemical composition of biominerals in plants (Franceschi and Nakata 2005; Rodríguez et al., 2005; He et al. 2012, 2014; De Giudici et al. 2017).

Waxes, covering leaf surface, consist predominantly of long-chain hydrocarbons, including alkanes, primary alcohols, aldehydes, secondary alcohols, ketones, esters, and other derived compounds. Currently, the classification of wax forms uses the Barthlott system (Dommisse et al., 2009; Koch et al. 2009). Three wax groups are identified: (1) biofilms (an amorphous thin layer) formed at the drying of epidermal structures that are not subject to cracking; (2) crusty layers which are thicker than biofilms and are characterized by an irregular surface and at the drying of the epidermis are fractured and break up into smaller pieces, they can be divided into three subgroups: smooth layer, shell layer, and cracked; and (3) crystalline forms as slabs, plates, tubes, pellets and filaments. Waxes leak onto the surface of the cuticle in the form of monomers and crystallize there.

Coal spoil heaps and heavy metal spoil heaps are very specific post-industrial habitats. Coal spoil heaps consist of carboniferous waste rock composed mainly of claystone, siltstone, sandstone, conglomerate, coal shale, and small quantities of coal (Rozkowski et al. 1993; Woźniak 2010). This substratum has unfavorable for plant growth properties, since it is characterized by low fertility, low water holding capacity (Li-ping et al. 2009), variable pH, high salinity, high temperatures, and low activity of microorganisms (Markowicz et al. 2015; Woźniak et al. 2015). Heavy metal spoil heaps are sites contaminated with heavy metals which are also characterized by low fertility, low water holding capacity, variable pH, and low activity of microorganisms (Gucwa-Przepióra et al. 2007; Niklińska and Stefanowicz 2015). In contrast to coal spoil heaps, however, heavy metals are the main factor which negatively affect growth of organisms on metal-contaminated spoil heaps (González and González-Chávez 2006; Gucwa-Przepióra et al. 2013; Wójcik et al. 2014; Bąba et al. 2016).

To date, the information on biomineralization in plants were mainly obtained from specimens which grew on natural or semi-natural habitats. As the biominerals reflect both the metabolic status of a vascular plant species and the environmental conditions of the plant habitat, it seems very interesting to find out which biominerals and waxes are present in/on leaves of plants growing on post-industrial sites. Thus, the aim of this study was to characterize biominerals occurring in the two plant species *Calamagrostis epigejos* and *Phragmites australis*, which are common grasses colonizing and growing on post-industrial sites. For investigations, we chose two habitats where both species occur abundantly on two different types of post-industrial sites and in an abandoned meadow site. In this way, it was possible to study biominerals and waxes present on individuals of the same species growing in different man-made habitats.

## Materials and methods

### Plant material

Small reed (*Calamagrostis epigejos*) is a plant species belonging to the *Poaceae* family. This species occurs naturally in Eurasia and Africa, but has now spread almost all over the globe (Kavanova and Gloser 2005). This grass was recorded from a range of habitats such as hay-meadows (Fiala et al. 2003), pastures (Stránská 2004), abandoned vineyards (Házi and Bartha 2002), and post-industrial and urban sites (Piekarska-Stachowiak et al. 2014; Stefanowicz et al. 2015). The post-abandonment spread of *C. epigejos* is facilitated by its ability to regenerate from rhizome fragments (Rebele and Lehmann 2001). It has very few habitat requirements. It is a photophilous species but also grows well in shaded areas. The water demand of this species is minimal and it can be considered an indicator of low groundwater levels. It also tolerates increased salinity and heavy metal content (Gloser et al. 2009).

Common reed (*Phragmites australis*) also is a species from the *Poaceae* family. It is widespread in wetlands throughout temperate and tropical regions around the world. In Europe, it grows mainly near stagnant water and oxbow lakes being the main element of rush vegetation around natural and anthropogenic water bodies. Recently, it has been frequently recorded in post-industrial and urban sites (Bartha et al. 2004; Woźniak 2005). This species is characterized by high tolerance to soil salinity (Vasquez et al. 2005) due to its large rhizomes which allows it to thrive in stressful environment (Lissner and Schierup 1997).

### Collection of plant samples

The leave samples of the studied grasses were collected from plants growing on three different sites: in post-industrial habitats—a coal spoil heap in Mysłowice a zinc spoil heap in Świętochłowice and a coal spoil heap in Katowice near Muchowiec airport all located in Upper Silesia in Southern Poland. In the preliminary study, vegetation patches with abundant occurrence of the studied grasses were selected. On each study site, three plots were established. Inside each plot five plant individuals were sampled and then from each plant the third leaf from the top was collected. For the microscopic observations, the middle part of the sampled leaves was used.

### The micro-structural observations

The micro-structural observations of the leaves and the micro-compositional analyses were conducted using a JSM-7600F scanning electron microscope (JEOL, Japan) equipped with EDX microprobe (Oxford, UK). Samples were mounted on the brass stubs with double sided carbon tape. Leaf samples which are non-conductive material were coated with a layer of gold having a thickness of 28 mm by using an ion coater (Quorum, UK). This metal coating of the sample allows to remove the excess of charge accumulating on the sample under the influence of the electron beam. The gold layer also protects the delicate tissue structure against the destructive energy of the electron beam (heat) concentrated on an extremely small area. The decisive factor is the relatively high thermal and electrical conductivity of metals which allows for rapid dissipation of energy. An additional effect of covering the biological sample with a heavy metal layer is to improve the electronic contrast by increasing the number of secondary and backscattered electrons. An accelerating voltage of 5–15 kV was used to view the specimens. Gold lines M _α_ 2.12 keV and M_β_ 2.20 keV overlap partly sulfur K_α_ 2.31 keV line in EDX spectra. However, the resolution of the used EDX is 130 eV so the S K_α_ is distinguishable.

## Results

**Fig. 1:**
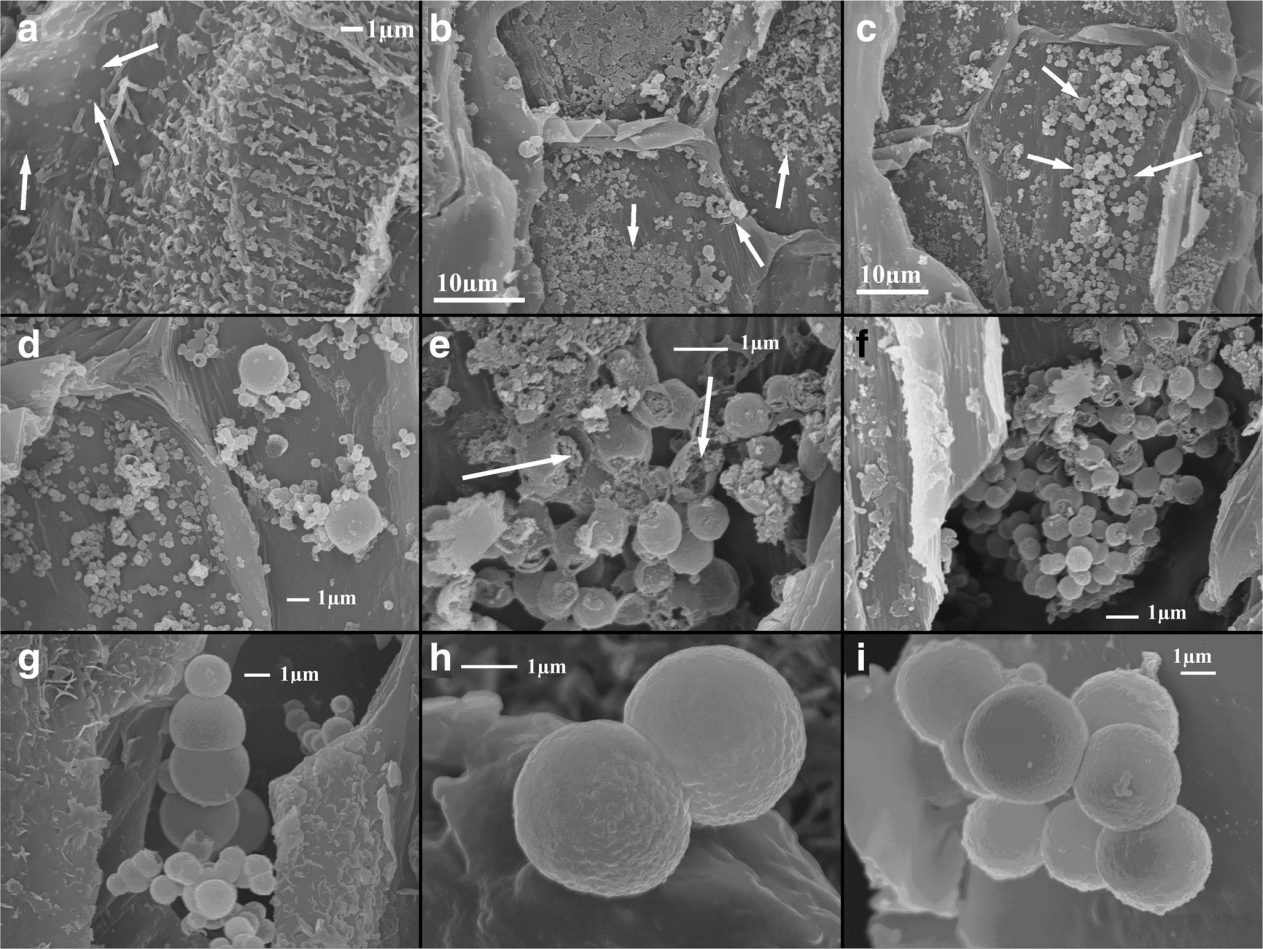
Development of calcium carbonate spherical forms in vascular bundles of *Calamagrostis epigejos*. **a** Precipitation of calcium carbonate in vascular bundles is shown by arrows. **b**, **c** Successive stages of agglomeration. **d** Spherulite formation in the Ostwald process. **e**, **f** Defected grown spheres. **g–i** In the final step, more perfect spheres are formed probably vaterite

The process of the formation of a calcium carbonate biomineral object in vascular bundles of *C. epigejos* is shown in Fig. 1. The leaf cross-section was obtained by fracturing with a scalpel. The secretion points of calcium carbonate are visible inside the vascular bundles in Fig. 1a. These secretions agglomerate (Fig. 1b) as a compact form. Next, the material self-organizes into sphere-like forms (Fig. 1c–f). The growing spheres become more perfect (Fig. 1g–i).

**Fig. 2:**
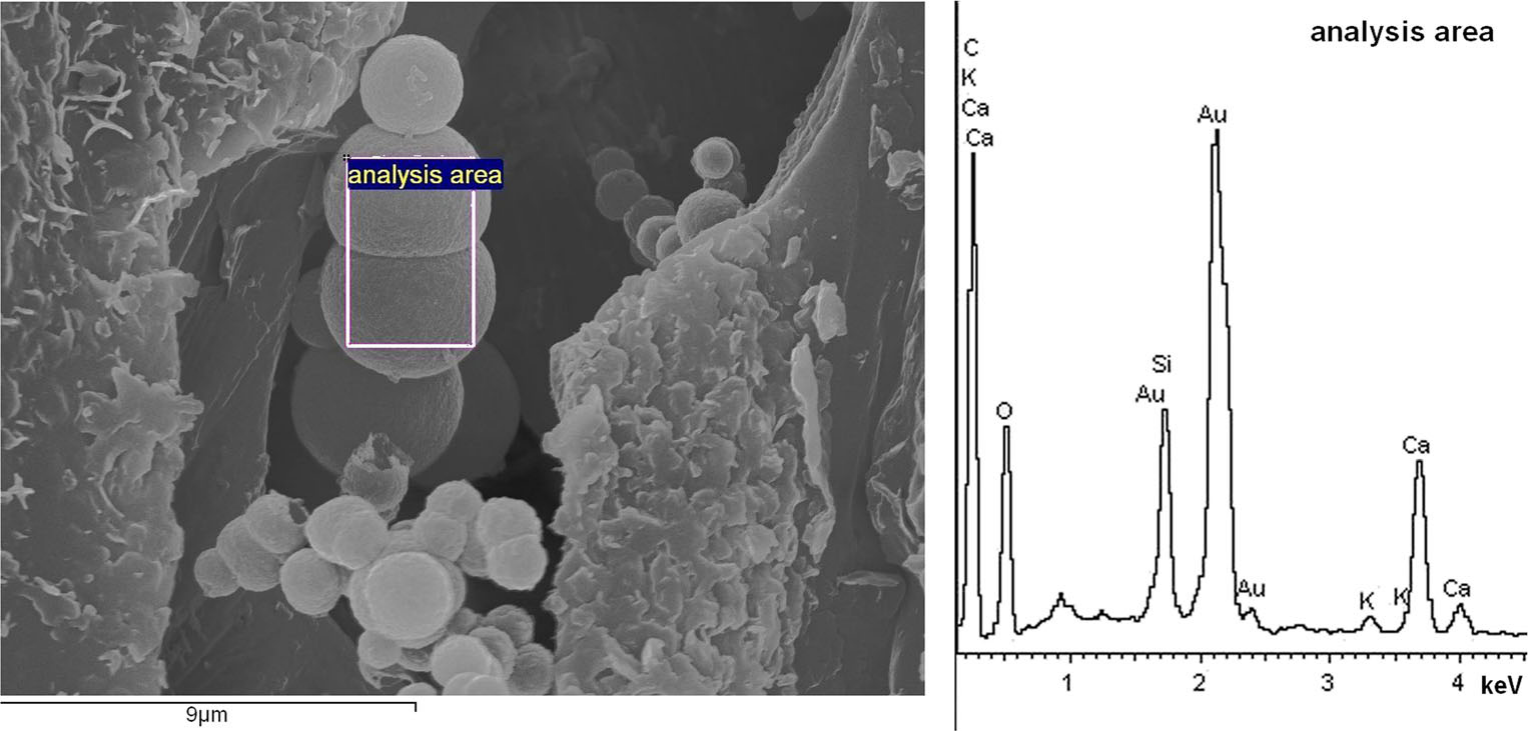
EDX analysis of the spherical forms shown in Fig. 1g. The sphere shape and chemical composition can indicate vaterite formation

The chemical analysis of spheres shown in Fig. 1g is presented in Fig. 2. Selected spheres contained Ca, C, O, and much less Si and K elements indicating the calcium oxalate or carbonate objects.

**Fig. 3:**
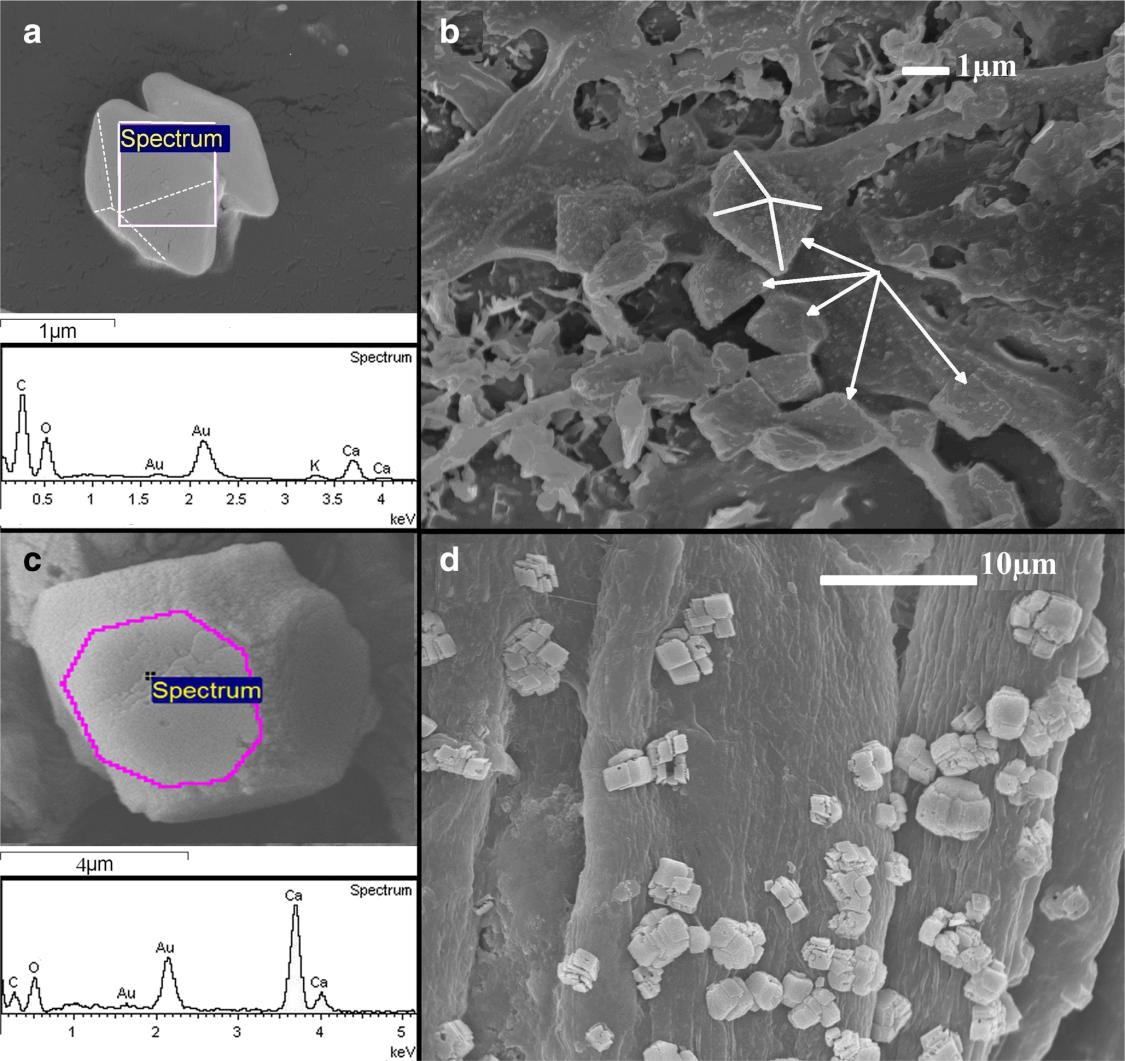
Calcium oxalate crystals in *Calamagrostis epigejos*. **a** Octahedral crystal the shape and chemical composition indicate calcium oxalate dehydrate. **b** Calcium crystals found on the surface of wax the octahedral shape indicates the calcium oxalate. **c** Calcium oxalate crystal obtained from leached leaf tissue. **d** Collection of leached crystals on the filter—many recesses visible on the crystals emerges due to removing organic matter by hydroxide

The examples of calcium oxalate crystalline forms are shown in Fig. 3. Figure 3a, b shows octahedrals of calcium oxalate dihydrate on *C. epigejos* leaves. Some leaves of *C. epigejos* were leached by using a saturated water solution of NaOH and the calcium compound objects in the sediment left on cellulose filter are shown in Fig. 3c–d both for *C. epigejos.* Figure 3c shows not fully shaped a crystal habit. The object visible in Fig. 3c has had its edges removed during leaching (Fig. 3d). It is suggested that they were built from organic materials (proteins and/or waxes) and were dissolved by NaOH solution (Holubowicz et al. 2015; Braissant et al. 2009). The chemical analysis by the EDX method gave a concentrations: Ca—11 at.%, C—41 at.%, O—48 at. % from a flat surface limited by a polygon visible in Fig. 3c. Such concentration could suggest calcium oxalate.

**Fig. 4:**
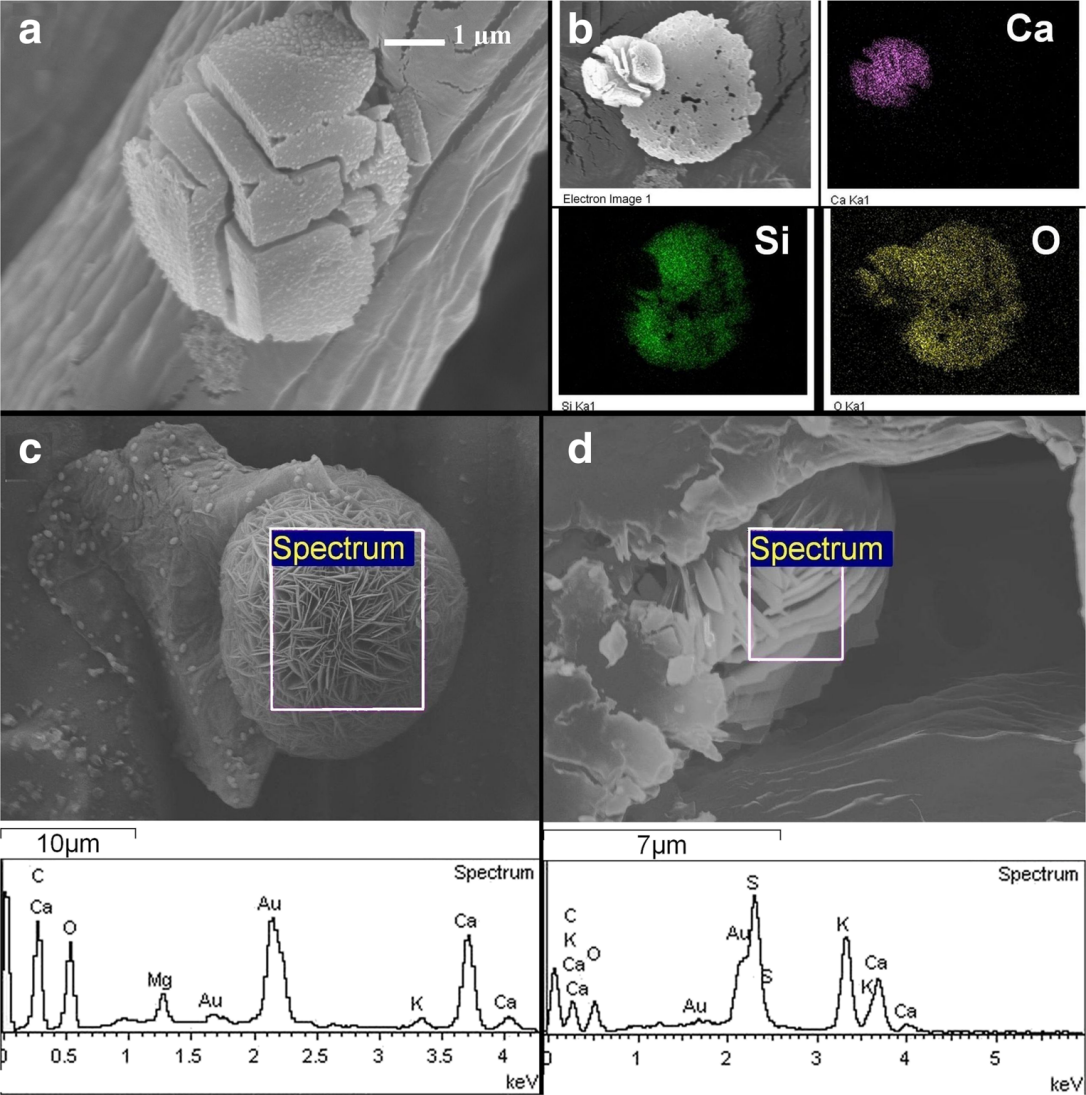
Calcium oxalate or carbonate crystals in *Phragmites australis*. **a** Leached crystal—many recesses visible on the crystals emerges due to removing organic tissue by hydroxide. **b** EDX analysis of leached calcium carbonate and silica phytolith. **c** Flower-like crystal built mainly from Ca with an admixture of Mg and K. **d** Flower-like calcium compound crystal with S and K found in vacuoles

More spectacular calcium carbonate or oxalate crystals were leached from *P. australis* leaves (Fig. 4). They are built from smaller parts of different shapes within the empty spaces of sites of organic matter. Micro-compositional analysis of objects shown in Fig. 4a reveals that cubic-like objects are built from Ca, C, and O (Fig. 4b). In Fig. 4b, besides cube-like mineral, the silica disc of phytolith is shown which is characteristic for *P. australis.* Valtchev et al. (2003) described process of zeolitization of silica objects in leaves of *Equisetum arvense* during hydrothermal treatment. We observed similar porous structure of phytolith after removing organic matter by NaOH leaching (Fig. 4b). Other forms of calcium compound crystals in the shape of 3d flowers were observed for both species and are presented in Fig. 4c–d.

**Fig. 5:**
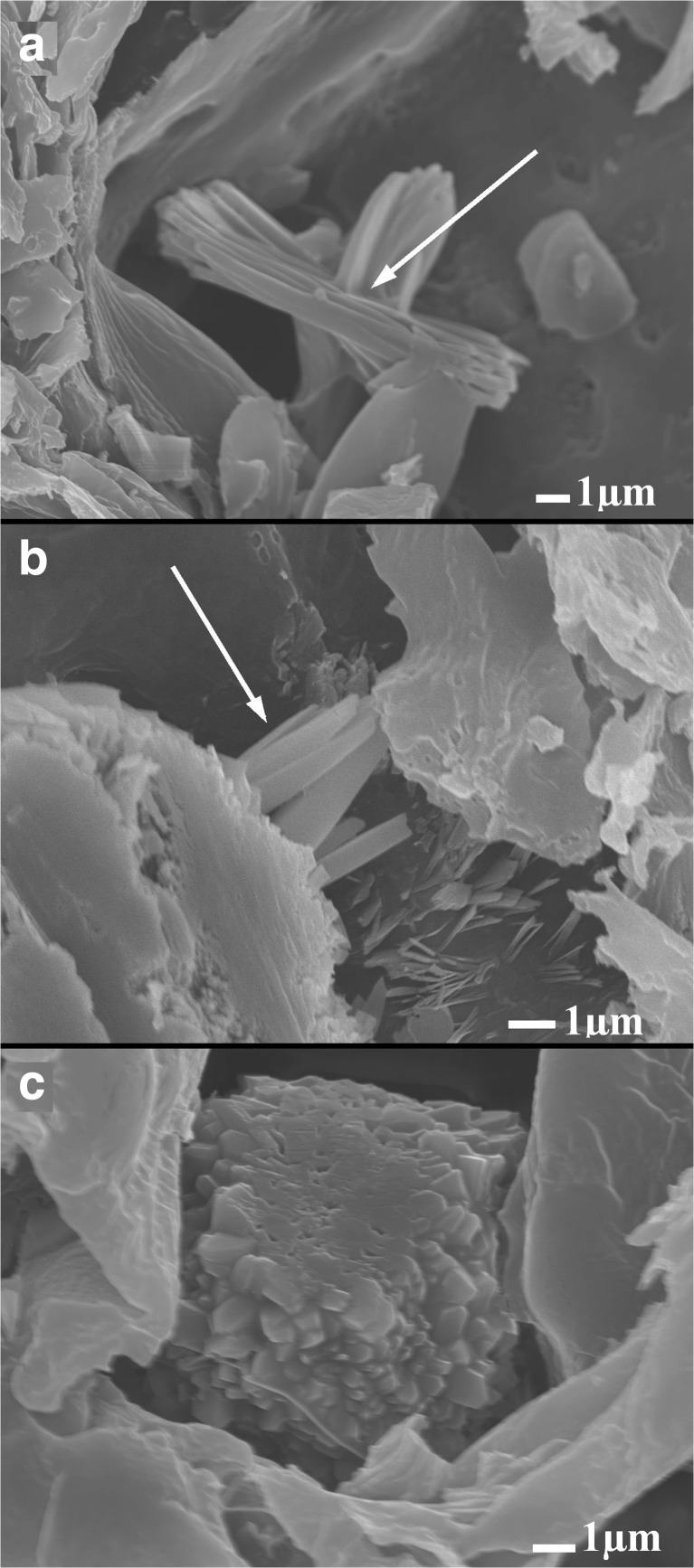
Typical forms of calcium oxalate crystals found in *Calamagrostis epigejos*. **a** Raphides. **b** Styloides. **c** Druses

The typical forms of calcium oxalate crystals such as raphides, styloides, and druses were also found in both plant species (Fig. 5).

**Fig. 6:**
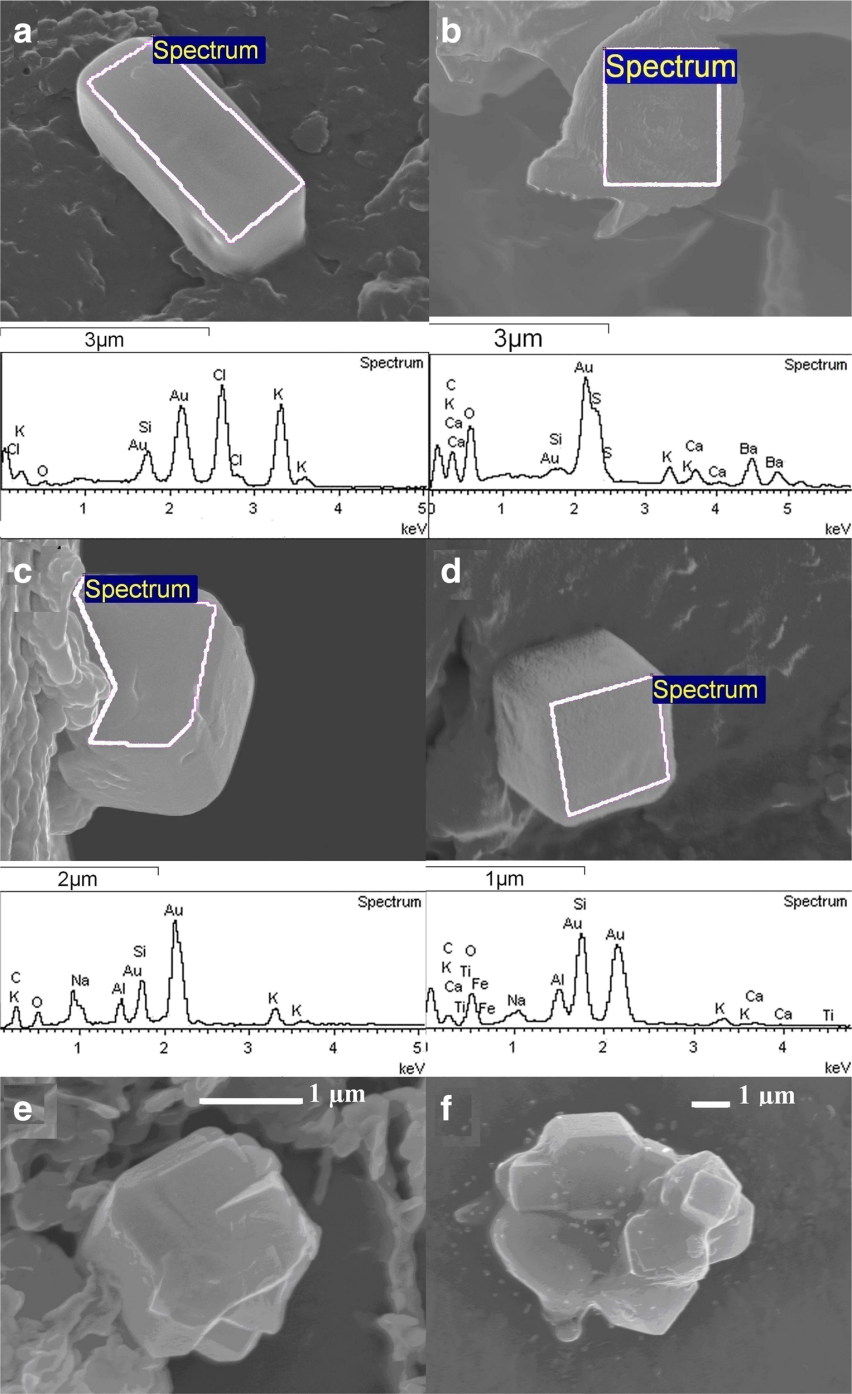
EDX analyses for mineral objects found on the leaves of *Calamagrostis epigejos*. All objects contain elements C and O coming from organic matter. Besides were observed. **a** Crystal rich in K Cl Si. **b** Crystal with Ba S K Si Ca. **c–f** Examples of crystals with similar composition having Al Si Ca with admixtures of another elements (Na, K, Fe, Ti)

The crystal of KCl is an example of another mineral observed on a *C. epigejos* leaf from the coal spoil heap in Katowice near Muchowiec airport. It is presented together with the elemental analysis (Fig. 6a). Figure 6b shows the object from Muchowiec site rich in barium sulfate whereas Fig. 6c–f show the different forms of crystals from plants collected on coal heap in Mysłowice.

**Fig. 7:**
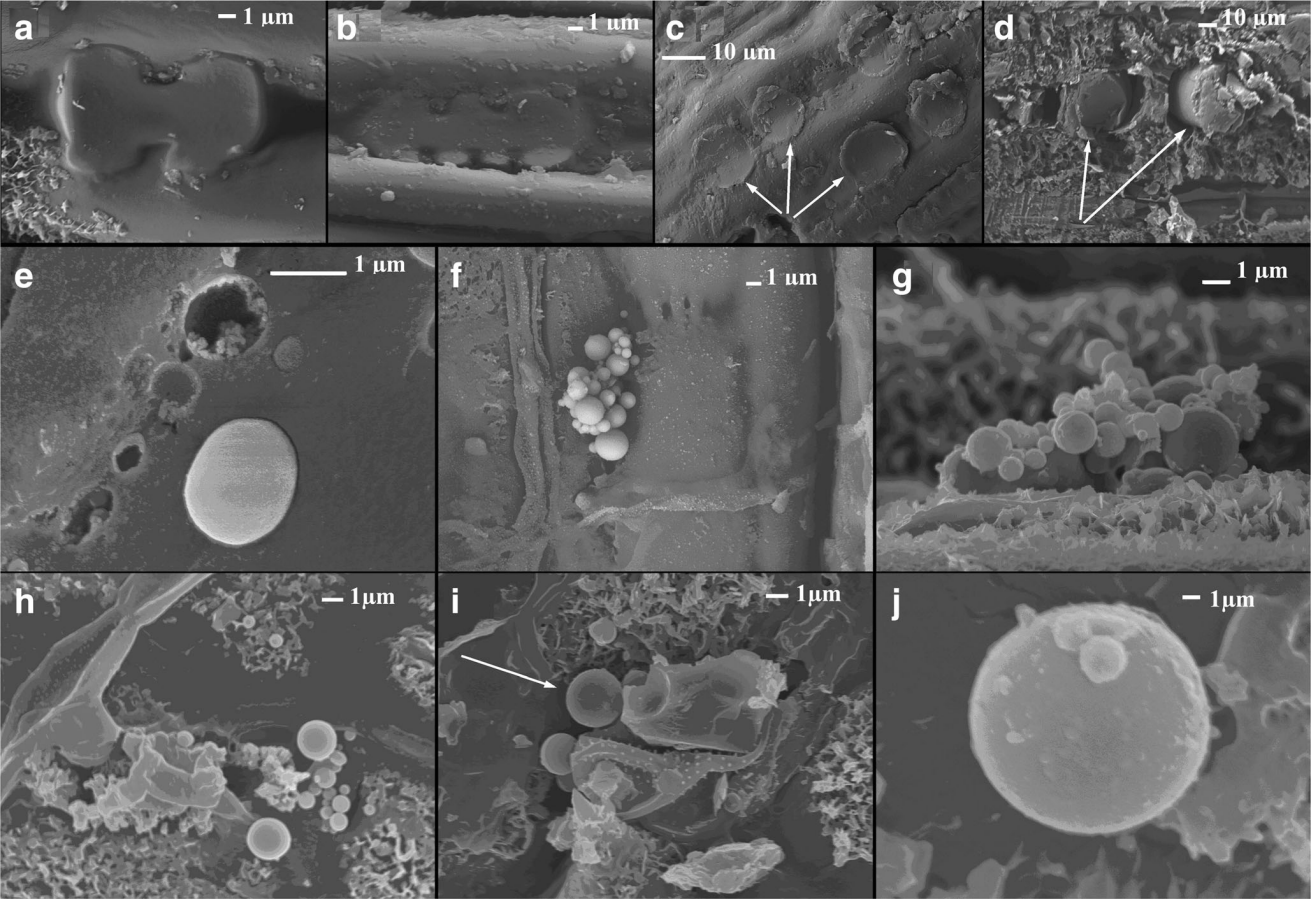
Silica objects. **a**, **b** Phytoliths of *Calamagrostis epigejos* of the deformed dumbbell form. **c** Phytoliths of *Phragmites australis*. **d** Phytoliths of *P. australis* as a result of force they have been dislocated in the leaf tissue. In this way, it was revealed that the phytolith is a cylinder. **e** Development of the silica forms for *C. epigejos*. **f**, **g** Silica balls for *C. epigejos*. **h–j** Aluminasilicate balls observed in *P. australis*

The examples of phytoliths in *P. australis* and *C. epigejos* are shown in Fig. 7a–d. In *P. australis* phytoliths were in the form of cylinders (Fig. 7a, b), whereas in *C. epigejos*, they were in the form of deformed dumbbells (Fig. 7c, d). In the present study, except phytoliths, some silica minerals were observed on the leaf surfaces. Moreover, some minerals were built not only from silica but they included additional elements as Al, K, Fe, Mn, Ti, etc. Such objects have been assigned to aluminosilicates. Figure 7e shows the development of ball-like forms of silica. A sand is created in mineralized wax enriched in silica (Fig. 7f, g). The sand agglomerates into larger ball-like objects. Silica forms predominate in *C. epigejos* (Fig. 7e–g) whereas aluminosilicates are mainly present in *P. australis* (Fig. 7h–j).

**Fig. 8:**
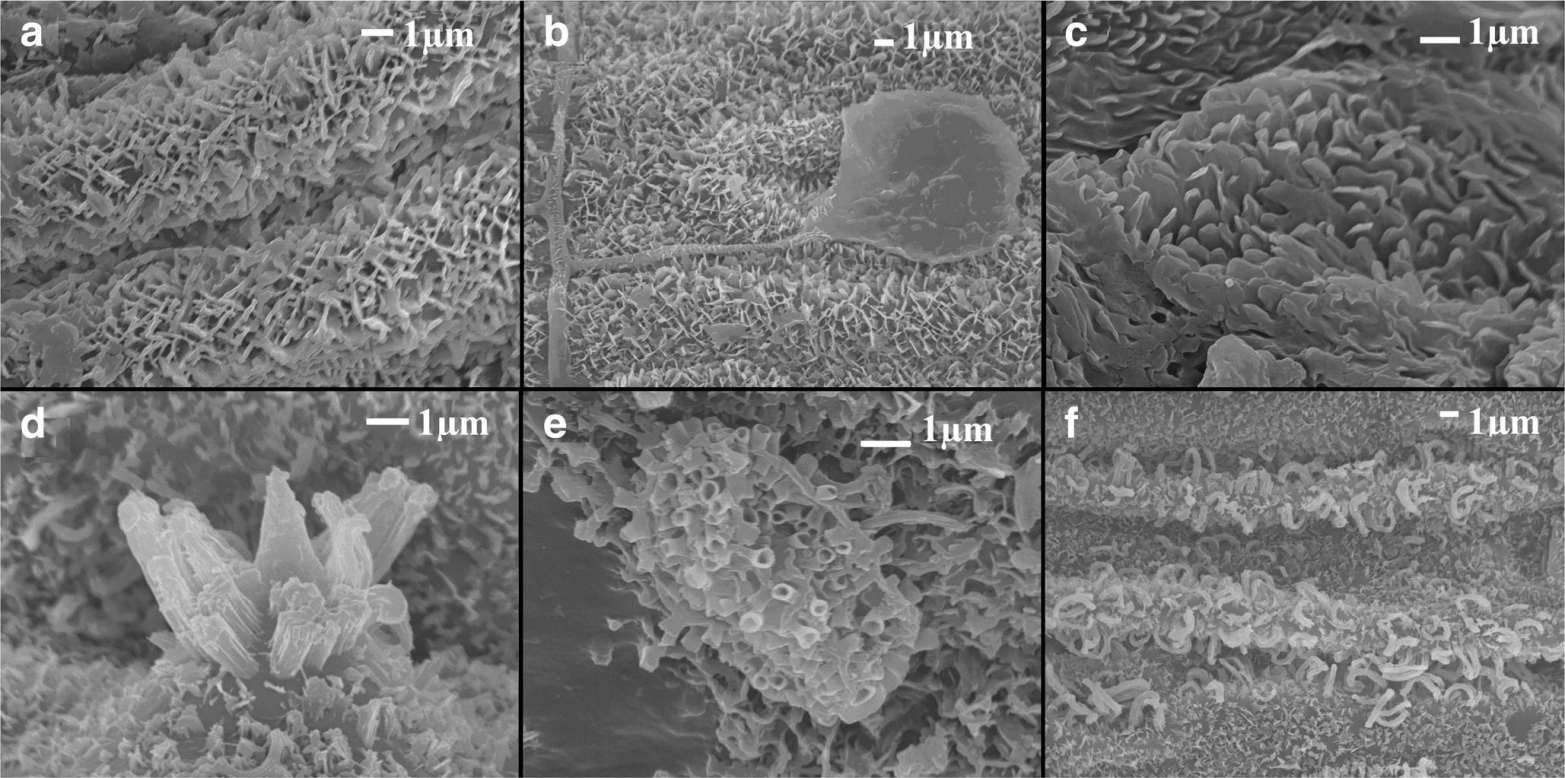
Waxes observed for *Calamagrostis epigejos*. **a** Randomly oriented plates. **b** Leakage of amorphous wax. **c** *Phragmites australis.* **d** Rodlets. **e** Tubes. **f** Curled forms

Many forms of waxes were observed (Fig. 8). For *C. epigejos* were observed on the examined species of plants: crystalline forms as plates (Fig. 8a, b), amorphous wax leakage (Fig. 8b), and flower like forms (Fig. 8c). Especially interesting are the tubules which were observed on *P. australis* leaves from the coal spoil heap (Fig. 8e). These tubular waxes are defined as cylindrical hollow crystals with an open end. They contain high concentrations of asymmetrical secondary alcohols (Riederer and Schreiber, 1995; Kunst and Samuels 2003; Yeats and Rose 2013). Moreover, the rodlets (Fig. 8d) and curled forms (Fig. 8f) were also observed (Jetter and Schäffer 2001; Dommisse et al., 2009; Koch et al. 2009).

## Discussion

Crystals of calcium carbonate and oxalate create a variety of structures. The presence of these crystals in higher plants is associated with the deposition of calcium ions (Franceschi and Nakata 2005; Dayod et al. 2010). A combination of genetic and environmental factors plays an important role in shaping the crystals of calcium compounds and in defining their functions within the plant. These crystals are formed in plants from an endogenous oxalic acid and calcium taken up from the soil. Oxalic acid (C_2_H_2_O_4_) is the simplest dicarboxylic organic acid and an oxidizing agent produced by the plant (Franceschi and Nakata 2005; He et al. 2014). According to studies on the deposition of calcium oxide crystals carried out on the leaves of *Morus alba* (mulberry plants), the number of calcium oxide crystals in the leaves decreased during calcium deficiency in the soil (Sugimura et al. 1999). This indicates that the accumulated element in the form of crystals can be used by plant during calcium deficiency (He et al. 2014).

Self-organization processes as presented in Fig. 1 were described in literature for amorphous calcium carbonate (ACC) (Rodriguez-Blanco et al. 2012; Bots et al. 2012). Disordered hydrated ACC forms from a highly supersaturated solution. The local order within the ACC increases concurrent with dehydration. The large difference in solubility between ACC and vaterite keeps the supersaturation at a sufficiently high level to allow continuous vaterite nucleation and spherulitic growth. Once initiated, spherulitic growth is maintained as long as highly soluble ACC is present in the system. Once all of the ACC has been consumed, the vaterite crystallite size continues to increase usually via Ostwald ripening. Because Ostwald ripening is a dissolution reprecipitation mechanism, the further ripening of the vaterite is easily displaced by a dissolution−reprecipitation transformation mechanism leading to the final calcite. Also, investigations of polymer-induced crystallization of ACC films and the sequential formation of CaCO_3_ structures assisted by the acidic polymer additives showing the similar behavior as in the present study were described by Cho (2017) and Ihli et al. (2014). They explained the formation of olive-like spheres in four stages. First, the balls built of ACC nanoparticles were observed. Then, surface crystallization of vaterite nanoparticles occurred. The diffusion of the Ca^2+^ and CO_3_^−2^ ions due to solubility of ACC forms vaterite spheres, which finally can partially collapse. Very similar mechanisms and objects were observed in the current study for *P. australis* and *C. epigejos*.

Holubowicz et al. (2015) crystallized calcium carbonate in the presence of Starmaker-like protein. They revealed that during the absence of protein calcium carbonate produced perfect rhombohedral crystals while those obtained in the presence of protein had a characteristic form with a stepped structure and had a characteristic rounding. Similar observations were made in *C. epigejos* (Fig. 3c). The leaf samples of *P. australis* from the post-industrial sites showed large amount of mineral forms even with the presence of sulfur aluminum and iron. The presence of significant amounts of carbon and oxygen and complicated forms in these biominerals may indicate the presence of organic nanofibres in their structure as indicated by recent studies (Kulak et al. 2015 Schulz et al. 2011).

The crystal of KCl is an example of another mineral observed on a *C. epigejos* leaf from the coal spoil heap in Katowice near Muchowiec airport. The presence of chlorides could be an effect of salinity of ground-waters which is characteristic for coal spoil heaps (Rozkowski and Rozkowski 1994). The presence of highly water soluble KCl crystals in plant tissue could be explained as a result of crystallization due to dehydration of plant material after harvesting. However, the presence of KCl crystals was reported by Brizuela et al. (2007) in leaves of *Tradescantia pallida*, suggesting that such crystals can be formed and be durable. The presence of water soluble crystals in plant tissue could be also explained by formation of complex compounds containing KCl as well as calcium oxalate, calcium sulfate, magnesium oxalate, or silica as it was proposed by Brizuela et al. (2007) and He et al. (2012). Franco et al. (2012) also found calcium oxalate with admixture of potassium in *Nerium oleander*. On the basis of literature data, it is tempting to suggest that KCl crystals observed on a *C. epigejos* leaf is not a result of crystallization due to dehydration of plant material after harvesting but it is biologically induced. The presence of these crystals only in *C. epigejos* seems to support this suggestion; however, further research is needed.

The physiological functions of phytolith probably rely on co-precipitation of metals with Si ions. Recently, it was proven that the addition of silicon in the soil reduces the detrimental effect of aluminum and manganese on plant growth (Pilon-Smits et al. 2009). The deposition of solid silica with aluminum near the cells has been observed in many plant species. Therefore, it is an essential mechanism for the detoxification of plants and it can be related to binding heavy metals in the form of silica (Ma and Yamaji 2006). Many authors suggest that co-precipitation with silicon may also function in plants as a detoxification mechanism of different heavy metals, as Cd, Cu, or Zn (Neumann and de Figueiredo 2002; Adrees et al. 2015; De Giudici et al. 2015; Medas et al. 2015). Phytoliths have also many structural functions in plants. Their structural function is an increasing the stiffness and strength of the plant which often causes a significant additional effect of increasing the availability of sunlight. Also, many authors draw attention for herbivory protection role of phytoliths (Massey et al. 2006; Hunt et al. 2008; He et al. 2014). Phytoliths were found in *P. australis* and *C. epigejos*. Moreover, silica minerals and aluminosilicates of different forms were observed in both species. It is noteworthy that silica forms predominated in *C. epigejos* whereas aluminosilicates were mainly present in *P. australis.* Similar silica forms as the observed in this work were found in *Imperata cylindrica* the other grass species (Rufo et al. 2014) and in *P. australis* (Schaller et al. 2013; De Giudici et al. 2017).

For the time being, there is a dearth of data available concerning oxalate and/or silica crystals formation in *P. australis* (Liu et al. 2012; Schaller et al. 2013; De Giudici et al. 2017) and lack of data for *C. epigejos* although for other species from genus *Calamagrostis* few papers were published, e.g., phytoliths were described in *C. villosa* (Carnelli et al. 2002).

The waxes observed for *C. epigejos* are mainly crystalline plates or amorphous form while in case of *P. australis* mainly rodlets and tubes were found.

## Conclusion

Research on the two grass species *Phragmites australis* and *Calamagrostis epigejos* from post-industrial sites confirmed the presence of crystalline forms of calcium oxalate or carbonate as well as the occurrence of multi-mineral forms. This study identified the presence of the following types of crystals: prismatic cubic-like spherical raphides, styloides, druses, sand and forms as amorphous calcium carbonate (ACC). For the first time, wide range of crystal forms is presented for *C. epigejos.* The leaf samples of *P. australis* from the post-industrial sites showed large amount of mineral forms even with the presence of sulfur aluminum and iron. It seems that waxes for both grasses show species dependence.

## References

[CR1] Adrees M, Ali S, Rizwan M, Zia-ur-Rehman M, Ibrahim M, Abbas F, Farid M, Farooq Qayyum M, KashifIrshad M (2015). Mechanisms of silicon-mediated alleviation of heavy metal toxicity in plants: a review. Ecotox Environ Safe.

[CR2] Bartha S, Campatella G, Canullo R, Bodis J, Mucina L (2004). On the importance of fine-scale spatial complexity in vegetation restoration. Int J Ecol Environ Sci.

[CR3] Bąba W, Błońska A, Kompała-Bąba A, Małkowski Ł, Ziemer B, Sierka E, Nowak T, Woźniak G, Besenyei L (2016). *Arbuscular mycorrhizal* fungi (AMF) root colonization dynamics of Molinia caerulea (L.) Moench. in grasslands and post-industrial sites. Ecol Eng.

[CR4] Bloom AJ, Smith S (2015) Mineral nutrition. In: Taiz L, Zeiger E, Møller IM, Murphy A. (eds.) Plant physiology and development. 6th ed. Sinauer Associates Inc. Sunderland Massachusettes USA 119–142

[CR5] Bots P, Benning LG, Rodriguez-Blanco JD, Roncal-Herrero T, Shaw S (2012). Mechanistic insights into the crystallization of amorphous calcium carbonate (ACC). Cryst Growth Des.

[CR6] Bouropoulos N, Weiner S, Addadi L (2001). Calcium oxalate crystals in tomato and tobacco plants: morphology and in vitro interactions of crystal-associated macromolecules. Chem – Europ J.

[CR7] Braissant O, Decho AW, Przekop KM, Gallagher KL, Glunk C, Dupraz C, Visscher PT (2009). Characteristics and turnover of exopolymeric substances in a hypersaline microbial mat. FEMS Microbiol Ecol.

[CR8] Brizuela M, Montenegro T, Carjuzaa P, Maldonado S (2007). Insolubilization of potassium chloride crystals in *Tradescantia pallida*. Protoplasma.

[CR9] Broadley M, Brown P, Cakmak I, Ma JF, Engel Z, Zhao F (2012) Beneficial elements. In: Marschner P. (ed.) Marschner's mineral nutrition of higher plants 3^rd^ ed. UK Elsevier 249–269

[CR10] Carnelli AL, Madella M, Theurillat J-P, Ammann B (2002). Aluminum in the opal silica reticule of phytoliths: a new tool in palaeoecological studies. Am J Bot.

[CR11] Cho K (2017) Polymer induced crystallization of amorphous CaCO_3_. http://crg.postech.ac.kr/korean/viewforum.php?f=18&sid=52fa299cfb1671253db91c0-dfe85f86f#name3. Accessed 3 Feb 2017

[CR12] Dayod M, Tyerman SD, Leigh RA, Gilliham M (2010). Calcium storage in plants and the implications for calcium biofortification. Protoplasma.

[CR13] De Giudici G, Medas D, Meneghini C, Casu MA, Gianoncelli A, Iadecola A, Podda S, Lattanzi P (2015). Microscopic biomineralization processes and Zn bioavailability: a synchrotron-based investigation of *Pistacia lentiscus* L. roots. Environ Sci Pollut Res.

[CR14] De Giudici G, Pusceddu C, Medas D, Meneghini C, Gianoncelli A, Rimondi V, Podda S, Cidu R, Lattanzi P, Wanty RB, Kimball BA (2017). The role of natural biogeochemical barriers in limiting metal loading to a stream affected by mine drainage. Appl Geochem.

[CR15] Dommisse A, Niemietz A, Barthlott W, Wandelt K, Koch K (2009). Nanostructure of epicuticular plant waxes: self-assembly of wax tubules. Surf Sci.

[CR16] Fiala K, Holub P, Sedláková I, Tuma I, Záhora J, Tesarová M (2003). Reason and consequences of expansion of *Calamagrostis epigejos* in alluvial meadows of landscape affected by water control measures—a multidisciplinary research. Ekol (Bratislava).

[CR17] Franceschi VR, Nakata PA (2005). Calcium oxalate in plants: formation and function. Annu Rev Plant Biol.

[CR18] Franco A, Rufo L, de la Fuente V (2012). Metal concentration and distribution in plant tissues of *Nerium oleander* (Apocynaceae, Plantae) from extremely acidic and less extremely acidic water courses in the Río Tinto area (Huelva, Spain). Ecol Eng.

[CR19] Gal A, Hirsch A, Siegel S, Li C, Aichmayer B, Politi Y, Fratzl P, Weiner S, Addadi L (2012). Plant cystoliths: a complex functional biocomposite of four distinct silica and amorphous calcium carbonate phases. Chem – Europ J.

[CR20] Gloser V, Košvancova M, Gloser J (2009). Changes in growth parameters and content of N-storage compounds in roots and rhizomes of *Calamagrostis epigejos* after repeated defoliation. Biol (Bratislava).

[CR21] González RC, González-Chávez MCA (2006). Metal accumulation in wild plants surrounding mining wastes. Environ Pollut.

[CR22] Gucwa-Przepióra E, Błaszkowski J, Kurtyka R, Małkowski Ł, Małkowski E (2013). *Arbuscular mycorrhiza* of *Deschampsia cespitosa* (*Poaceae*) at different soil depth in highly metal-contaminated site in southern Poland. Acta Soc Bot Pol.

[CR23] Gucwa-Przepióra E, Małkowski E, Sas-Nowosielska A, Kucharski R, Krzyżak J, Kita A, Römkens PFAM (2007). Effect of chemophytostabilization practices on arbuscular mycorrhiza colonization of *Deschampsia cespitosa* ecotype Waryński at different soil depths. Environ Pollut.

[CR24] Hawkesford H, Horst W, Kichey T, Lambers H, Schjoerring J, Skrumsager-Möller I, White P (2012) Functions of macronutrients. In: Marschner P. (ed.), Marschner’s mineral nutrition of higher plants 3^rd^ ed. UK Elsevier 135–189

[CR25] Házi J, Bartha S (2002) The role of *Calamagrostis epigejos* in the succession of abandoned vineyards in the Western Cserhát Hungary. 3rd European Conference on Restoration Ecology Conference Abstracts. pp.126

[CR26] He H, Bleby TM, Veneklaas EJ, Lambers H, Kuo J (2012). Morphologies and elemental compositions of calcium crystals in phyllodes and branchlets of *Acacia robeorum* (*Leguminosae: Mimosoideae*). Ann Bot.

[CR27] He H, Kirilak Y, Kuo J, Lambers H (2015). Accumulation and precipitation of magnesium calcium and sulfur in two *Acacia* (*Leguminosae; Mimosoideae*) species grown in different substrates proposed for mine-site rehabilitation. Am J Bot.

[CR28] He H, Veneklaas EJ, Kuo J, Lambers H (2014). Physiological and ecological significance of biomineralization in plants. Trends Plant Sci.

[CR29] Holubowicz R, Porębska A, Poznar M, Różycka M, Dobryszycki P (2015). Biomineralisation—precision of shape structure and properties controlled by proteins. Post Biochem.

[CR30] Hunt JW, Dean AP, Webster RE, Johnson GN, Ennos AR (2008). A novel mechanism by which silica defends grasses against herbivory. Ann Bot.

[CR31] Ihli J, Wong WC, Noel EH, Kim Y-Y, Kulak AN, Christenson HK, Duer MJ, Meldrum FC (2014). Dehydration and crystallization of amorphous calcium carbonate in solution and in air. Nat Commun.

[CR32] Jetter R, Schäffer S (2001) Chemical composition of the *Prunus laurocerasus* leaf surface. Dynamic changes of the epicuticular wax film during leaf development. Plant Physiol 126:1725–1737 10.1104/pp.126.4.1725 PMC117171 11500570

[CR33] Jungk AO (2002) Dynamics of nutrient movement at the soil-root interface. In: Waisel Y Eshel A Kafkafi U. eds. Plant Roots. The hidden half 3^rd^ ed. USA: Marcel Dekker Inc. 587–616

[CR34] Kabata-Pendias A (2011) Trace elements in soils and plants. 4^th^ ed. CRC Press Taylor and Francis Group Boca Raton USA

[CR35] Kavanova M, Gloser V (2005). The use of internal nitrogen stores in the rhizomatous grass *Calamagrostis epigejos* during regrowth after defoliation. Ann Bot.

[CR36] Koch K, Bhushan B, Barthlott W (2009). Multifunctional surface structures of plants. Prog Mater Sci.

[CR37] Kulak A, Yang P, Semsarilar M, Cespedes O, Kim YY, Armes SP, Meldrum FC (2015) Bio-inspired composite crystals. Incorporation of nanoparticles in calcite and zinc oxide single crystal. Conference lecture at ECCG 5 8–11 September 2015 Bologna Italy

[CR38] Kunst L, Samuels AL (2003). Biosynthesis and secretion of plant cuticular wax. Prog Lipid Res.

[CR39] Lersten NR (1983). Crystals of calcium compounds in *Gramineae*. New Phytol.

[CR40] Li-ping W, Kui-mei Q, Shi-long H, Bo F (2009). Fertilizing reclamation of *arbuscular mycorrhizal* fungi on coal mine complex substrate. Proced Earth Plan Sc.

[CR41] Lissner J, Schierup H (1997). Effects of salinity on the growth of *Phragmites australis*. Aquat Bot.

[CR42] Liu Y, Li X, Liu M, Cao B, Tan H, Wang J, Li X (2012). Responses of three different ecotypes of reed (*Phragmites communis Trin.*) to their natural habitats: leaf surface micro-morphology anatomy chloroplast ultrastructure and physio-chemical characteristics. Plant Physiol Biochem.

[CR43] Ma JF, Yamaji N (2006). Silicon uptake and accumulation in higher plants. Trends Plant Sci.

[CR44] Markowicz A, Woźniak G, Borymski S, Piotrowska-Seget Z, Chmura D (2015). Links in the functional diversity between soil microorganisms and plant communities during natural succession in coal mine spoil heaps. Ecol Res.

[CR45] Massey FP, Ennos AR, Hartley SE (2006). Silica in grasses as a defence against insect herbivores: contrasting effects on folivores and a phloem feeder. J Anim Ecol.

[CR46] McConn MM, Nakata PA (2002). Calcium oxalate crystal morphology mutants from *Medicago truncatula*. Planta.

[CR47] Medas D, De Giudici G, Casu MA, Musu E, Gianoncelli A, Iadecola A, Meneghini C, Tamburini E, Sprocati AR, Turnau K, Lattanzi P (2015). Microscopic processes ruling the bioavailability of zn to roots of *Euphorbia pithyusa* L. pioneer plant. Environ Sci Technol.

[CR48] Mengel K, Kirkby EA, Kosegarten H, Appel T (2001) Principles of plant nutrition. fifth ed. Kluwer Academic Publishers Dordrecht The Netherlands

[CR49] Nakata PA (2012). Plant calcium oxalate crystal formation function and its impact on human health. Front Plant Biol.

[CR50] Neumann D, De Figueiredo C (2002). A novel mechanism of silicon uptake. Protoplasma.

[CR51] Niklińska M, Stefanowicz AM (2015) Soil microorganisms in heavy metal contaminated sites. In: Wierzbicka M ed. Ecotoxicology. Plants soils metals. Warsaw University Warsaw Poland 207–225. **(in Polish)**

[CR52] Piekarska-Stachowiak A, Szary M, Ziemer B, Besenyei L, Woźniak G (2014). An application of the plant functional group concept to restoration practice on coal mine spoil heaps. Ecol Res.

[CR53] Pilon-Smits EAH, Quinn CF, Tapken W, Malagoli M, Schiavon M (2009). Physiological functions of beneficial elements. Curr Opin Plant Biol.

[CR54] Piperno DR (2006) Phytoliths a comprehensive guide for archeologists and paleoecologists. AltaMira Press 2006 Lanhamm MD USA

[CR55] Prychid CJ, Rudall PJ (1999). Calcium oxalate crystals in monocotyledons: a review of their structure and systematics. Ann Bot.

[CR56] Rebele F, Lehmann C (2001). Biological flora of Central Europe: *Calamagrostis epigejos* (*L*.) *Roth*. Flora.

[CR57] Riederer M, Schreiber L (1995) Waxes: the transport barriers of plant cuticles. In: RJ Hamilton ed Waxes: Chemistry Molecular Biology and Functions. Oily Press Dundee UK 131–156

[CR58] Rodríguez N, Menéndez N, Tornero J, Amils R, de la Fuente V (2005). Internal iron biomineralization in *Imperata cylindrica*, a perennial grass: chemical composition, speciation and plant localization. New Phytol.

[CR59] Rodriguez-Blanco JD, Shaw S, Bots P, Roncal-Herrero T, Benning LG (2012). The role of pH and Mg on the stability and crystallization of amorphous calcium carbonate. J Alloy Compd.

[CR60] Rozkowski A, Chmura A, Gajowiec B, Wagner J (1993). Impact of mining on the groundwater chemistry in the Upper Silesian Coal Basin (Poland). Mine Water Environ.

[CR61] Rozkowski A, Rozkowski J (1994) Impact of mine waters on river water quality in the Upper Silesian coal basin 5th International Mine Water Congress Nottingham (U.K.) Proceedings 811–821

[CR62] Rufo L, Franco A, de la Fuente A (2014). Silicon in *Imperata cylindrica* (L.) P. Beauv: content distribution and ultrastructure. Protoplasma.

[CR63] Sarret G, Isaure M-P, Marcus MA, Harada E, Choi Y-E, Pairis S, Fakra S, Manceau A (2007). Chemical forms of calcium in Ca, Zn- and Ca, Cd containing grains excreted by tobacco trichomes. Can J Chem.

[CR64] Schulz A, Wang H, van Rijn P, Boeker A (2011). Synthetic inorganic materials by mimicking biomineralization processes using native and non-native protein functions. J Mater Chem.

[CR65] Schaller J, Brackhage C, Paasch S, Brunner E, Bäucker E, Gert Dudel E (2013). Silica uptake from nanoparticles and silica condensation state in different tissues of *Phragmites Australis*. Sci Total Environ.

[CR66] Skinner HCW, Jahren AH (2003). Biomineralization. Treat Geochem.

[CR67] Stefanowicz AM, Kapusta P, Błońska A, Kompała-Bąba A, Woźniak G (2015). Effects of *Calamagrostis epigejos*, *Chamaenerion palustre* and *Tussilago farfara* on nutrient availability and microbial activity in the surface layer of spoil heaps after hard coal mining. Ecol Eng.

[CR68] Stránská M (2004). Successional dynamics of *Cynosurus pasture* after abandonment in Podkronoší. Plant Soil Environ.

[CR69] Sugimura Y, Mori T, Nitta I, Kotani E, Furusawa T, Tatsumi M, Kusakari SI, Wada M, Morita Y (1999). Calcium deposition in idioblasts of mulberry leaves. Ann Bot.

[CR70] Valtchev V, Smaihi M, Faust A-C, Vidal L (2003). Biomineral-silica-induced zeolitization of *Equisetum Arvense*. Angew Chem Int Ed.

[CR71] Vasquez EA, Glenn EP, Brown JJ, Guntenspergen GR, Nelson SG (2005). Salt tolerance underlies the cryptic invasion of North American salt marshes by an introduced haplotype of the common reed *Phragmites australis* (*Poaceae*). Mar Ecol-Prog Ser.

[CR72] White PJ, Broadley MR (2003). Calcium in plants. Ann Bot.

[CR73] Woźniak G (2005) Problems of *Calamagrostis epigejos* synecology on post-industrial sites. In: Biology of grasses. Edited by Frey L. W. Szafer Institude of Botany Polish Academy of Science Kraków pp. 353–361

[CR74] Woźniak G (2010) Diversity of vegetation on coal-mine heaps of the Upper Silesia (Poland). **Szafer Institute of Botany Polish Academy of Sciences Cracow Poland**. **(in Polish)**

[CR75] Woźniak G, Markowicz A, Borymski S, Piotrowska-Seget Z, Chmura D, Besenyei L (2015). The relationship between successional vascular plant assemblages and associated microbial communities on coal mine spoil heaps. Comm Ecol.

[CR76] Wójcik M, Sugier P, Siebielec G (2014). Metal accumulation strategies in plants spontaneously inhabiting Zn-Pb waste deposits. Sci Total Environ.

[CR77] Xu GX, Tan C, Wei XJ, Gao XY, Zheng HQ (2011). Development of secretory cells and crystal cells in *Eichhornia crassipes* ramet shoot apex. Protoplasma.

[CR78] Yeats TH, Rose JKC (2013). The formation and function of plant cuticles. Plant Physiol.

